# Development of a risk model based on immune genes in patients with colon adenocarcinoma

**DOI:** 10.1002/cnr2.1712

**Published:** 2022-09-04

**Authors:** Laiming Wei, Jing Xu, Xueyou Hu, Gang Lyu

**Affiliations:** ^1^ Department of Electronic and Information Engineering, School of Advanced Manufacturing Engineering Hefei University Hefei China; ^2^ Department of Oncology The First Affiliated Hospital of Anhui Medical University Hefei China; ^3^ School of Big data and Artificial Intelligence Chizhou University Chizhou Anhui China

**Keywords:** colon adenocarcinoma, immune genes, prognostic model, TCGA

## Abstract

**Background:**

The rapidly increasing morbidity and the poor prognosis making the colon adenocarcinoma not only common but also highly malignant. On the other hand, immunotherapy emerges as a therapeutic modality of colon cancer recently. In this study, we developed a prognostic risk model that is based on immune genes, which could predict the overall survival (OS) of patients with colon adenocarcinoma.

**Methods:**

The Cancer Genome Atlas (TCGA) database was used to download both transcriptomic and clinical data, and the ImmPort database was used to obtain immune genes. The least absolute shrinkage and selection operator (LASSO)‐Cox regression was adopted to further select the key genes with prognostic value. Then the key genes were inputted into stepwise regression to calculated each patient's immune‐related risk score (immune score). Survival, survminer packages and bilateral tests in R language were adopted to determine the optimal cut‐off value (cut‐off value) for the risk score. This threshold divides patients into immune‐score high‐risk and low‐risk groups. The differences in the levels of infiltrating immune cells and stromal cells in the high and low immune risk groups were then calculated and compared by the CIBERSORT algorithm.

**Results:**

According to our results, a prognostic risk model was constructed based upon 26 immune‐related genes. High immune score was shown to be a poor prognostic factor for colon adenocarcinoma patients, such as overall survival, progress free survival for different therapies, and tumor stages. High immune score was also associated with the abundance of CD4+ T cells and CD8+ T cells. In addition, the high immune score group, the expression levels of LMTK3, LAG3 and PD‐L1 were higher than those in the low score group.

**Conclusion:**

We developed a 26‐immune gene model of colon adenocarcinoma to predict patient's survival. This model might be used in clinical practice as a prognostic instrument for patients diagnosed with colon adenocarcinoma.

## 1 INTRODUCTION

Colon adenocarcinoma is the third most common malignancy worldwide and the second leading cause of cancer‐related death.^1^ Surgery is considered the primary therapeutic strategy for the management of colon adenocarcinoma, as well as systemic therapy. However, approximately 25%–40% of patients have postoperative recurrence and poor prognosis.^2^ At present, the tumor‐lymph‐node‐metastasis (TNM) stage system is considered as a standard classification to predict the survival and prognosis in patients with colon adenocarcinoma.^3^ Colon adenocarcinoma is widely regarded as a disease with clinical heterogeneity and immunogenicity. So it is difficult to accurately assess the prognosis of each patient if only TNM stage system is adopted. Therefore, it is a great challenge to predict the survival rate of colon adenocarcinoma patients accurately and individually, as well as prolong their survival.

The immune system is important in cancer development, progression and metastasis.^4^ Colon cancer treatment strategies have become increasingly sophisticated and have developed into a new stage.^5^ Immunotherapy is a new type of cancer therapy, which targets the human immune system. It was also found that lack of T‐cell infiltration predicted poor outcomes in colorectal cancer patients.^6^ More and more studies have found that numerous immune‐related genes are related to the occurrence and development of colon adenocarcinoma.^7^, ^8^ Although it was shown that colon cancer with microsatellite instability might benefit from immunotherapy,^4^ PD‐1/‐L1 inhibitors or CTLA 4 inhibitors were not reported to have efficacy in patients with unselected colon cancer.^9^ Thus, it is important to develop a prognostic model for multiple immune genes to reflect the sensitivity of patients to immunotherapy.

In this study, we selected the differentially expressed immune‐related genes from The Cancer Genome Atlas (TCGA) and ImmPort databases. The immune‐related DEGs with prognostic value were identified according to bioinformatics analyses. The key immune genes were screened to construct a risk model for patients with colon adenocarcinoma.

## 2 MATERIALS AND METHODS

### 2.1 Data acquisition

We downloaded the RNA sequencing data colon adenocarcinoma samples and their relevant clinical information from The Cancer Genome Atlas (TCGA) database (https://portal.gdc.cancer.gov). The colon adenocarcinoma dataset included 459 tumor samples and 41 adjacent normal samples. After careful survey, samples from patients with survival time less than 30 days or insufficient clinical data were excluded. Four hundred and sixteen samples with integral clinical information were included in the follow‐up analysis finally. AffymetrixHuman Genome U133 Plus 2.0 Array platform was utilized to analyze the samples. Log2 (fragments per kilobase of exon model per million mapped fragments [FPKM] + 0.01) was used to normalize he gene expression. Due to the access policies of TCGA program, all data from TCGA websites are available publicly. Therefore, our study did not need Ethics Committee approval. In addition, a comprehensive list of 2483 immune‐related genes were obtained from ImmPort database (https://www.immport.org).

### 2.2 Differential gene analysis

The differential expression genes (DEGs) between tumor tissues and adjacent normal tissues were analyzed using the R language's limma function package (R version 3.5.2). We used the absolute values of differential expression multiples (Log2FC) of logarithmic transformation >1 and FDR ≤0.05 as criteria to select DEGs. Immune‐related DEGs were identified using Venny diagram (Venny 2.1.0).

### 2.3 Construction of the prognostic immune score model

Immune genes with prognostic value were firstly screened out using univariate Cox regression analysis with *p* < .05 as a threshold. Schoenfeld residuals test were performed to determine the adequacy of the PH assumptions (Section S1 in Supplemental Material, Figure S1). Least absolute shrinkage and selection operator (LASSO) Cox regression analysis (cv.glmnet package) was performed to select the key immune genes and develop a prognostic risk model to minimize the level of overfitting.^10^ We calculated the Risk Score (immune score) of each patient based upon the key immune genes according to the following formula.

$$\text{immune score}=\sum_{i=1}^{n}+{\text{Coef}}_{i}*X_{i}$$

Coefi was the risk coefficient of each gene expression from the LASSO‐Cox model. And $X_{i}$ was the expression value of key immune gene. Regression analysis was performed on immune score and survival time (Section S2 in Supplemental Material). Our data satisfy the three conditions of independence, homogeneity of variance and normality, which indicated that immune score met the conditions of linear regression independence.

Next, survival, survminer, and bilateral test of R package were performed to determine the optimal cutoff value of the Risk Scores. Thus, the patients were classified into two risk groups: high immune score group and low immune score group.

Time‐dependent subject operating work characteristics (ROC) curves were drawn by R language survival ROC package. And we adopted the multivariate Cox model to determine whether our Risk Score model could predict the overall survival of patients independently.

### 2.4 Functional enrichment analysis

Enrichment analyses were carried out for the key immune genes using “clusterProfiler” function package in R, including biological process (BP), molecular function (MF), cellular component (CC) and Kyoto Encyclopedia of Genes and Genomes (KEGG) Pathway enrichment analysis. *p* Value less than .05 was considered statistical significance.

### 2.5 Treatment sensitivity and immune infiltration in patients with various immune scores

TCGA database provided therapeutic information of included samples. The progression‐free survival (PFS) of different treatments between different immune score groups was compared through the Kaplan–Meier analysis with log‐rank tests. The cMAP database (http://www.complement.us/labweb/cmap) assesses the sensitivity of the high immune risk group to chemotherapy drugs in HT29 human intestinal cancer cell line. In addition, different levels of infiltrating immune cells and stromal cells in high and low immune risk groups were analyzed by CIBERSORT algorithms (https://cibersort.stanford.edu).

### 2.6 Statistical analyses

Student's *t*‐test was utilized to compare gene expression differences between tumor and adjacent normal tissues. The Spearman's method was applied to study the correlation coefficient. The OS between different groups was accessed by the chi‐squared test and Fisher's exact test. Univariate Cox and multivariate Cox regression analyses were performed to identify independent survival predictors. R software (Version 3.5.3) was used to conduct all statistical analyses. *p* < .05 was considered as statistically significance. And *p* values were two‐tailed.

## 3 RESULTS

### 3.1 Identification of immune‐related DEGs with prognostic values in the TCGA colon adenocarcinoma cohort

Figure S5 showed the flow chart of our work. There were 415 colon cancer samples included in this study. The mean age of them was 66 years old. 46.4% of them were female patients and 53.6% were males. 40% of the colon cancer occurred in left colon. In addition, about half of the patients were diagnosed with stage I/II colon cancer and half were stage III/IV colon cancer.

**FIGURE 1:**
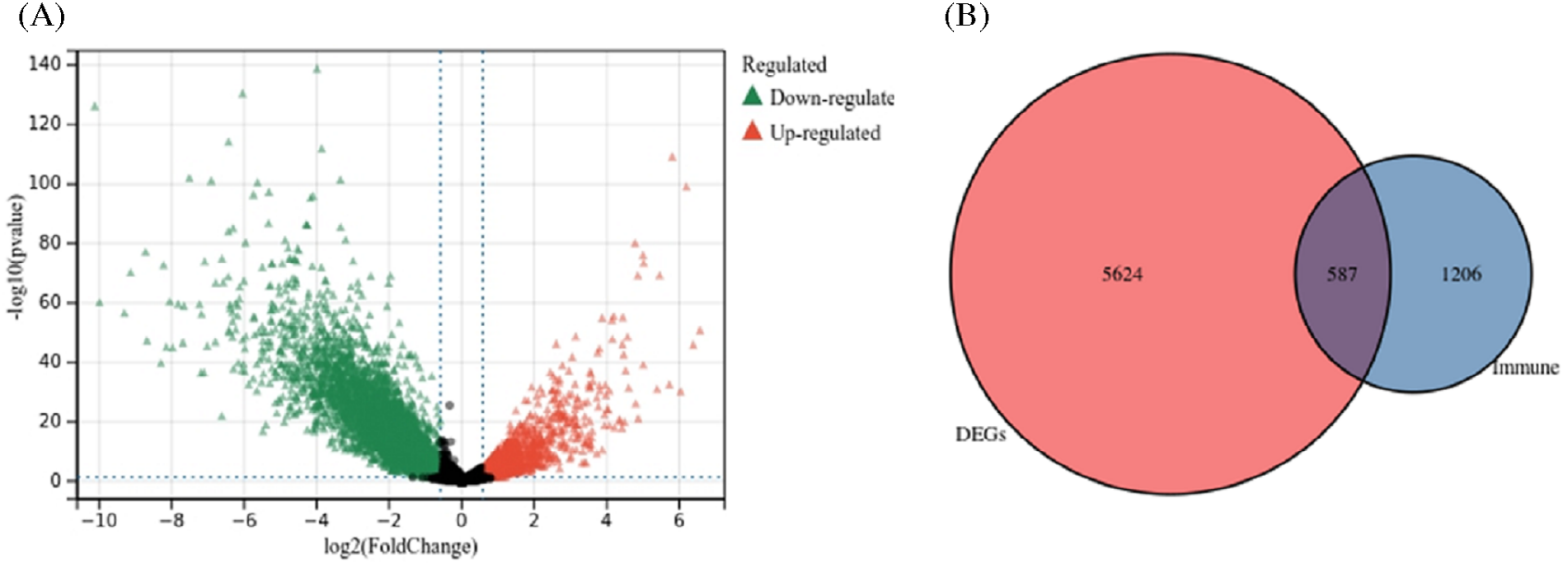
Identification of prognostic immune‐related differential expression genes (DEGs). (A) Volcano plot of DEGs between tumor samples and normal tissue samples, (B) Venn diagram to identify immune‐related DEGs

A total of 6203 DEGs between tumor samples and normal samples were identified in the TCGA colon adenocarcinoma cohort (Figure 1A). Among them, 587 DEGs were found to be immune related genes (Figure 1B). Univariate Cox regression analysis revealed that 46 immune genes were closely related to colon adenocarcinoma prognosis (*p* < .05; Table 1). In tumor tissues, higher expression of 34 immune genes predicted worse OS in patients with colon adenocarcinoma (Table 1).

**TABLE 1:** The results of univariate Cox regression analysis between the expression of 46 candidate immune‐related genes and OS for colon adenocarcinoma

Genes	HR	95% CI lower	95% CI upper	*Z*	*p* Value
sHSPA1A	0.231772	0.100168	0.363377	3.451743	5.57E‐04
UCN	0.316834	0.128604	0.505065	3.299057	9.70E‐04
PTH1R	0.228944	0.084963	0.372924	3.116533	0.00183
MCHR2	0.398299	0.135385	0.661213	2.969232	0.002985
NGF	0.239939	0.080918	0.39896	2.95729	0.003104
LEP	0.14721	0.048191	0.246229	2.913858	0.00357
PLXNB3	0.152644	0.041229	0.264059	2.685247	0.007248
TDGF1	−0.10849	−0.18964	−0.02735	−2.62055	0.008779
NOX5	0.226436	0.053742	0.399129	2.569908	0.010173
IFNE	0.151318	0.035793	0.266843	2.567212	0.010252
SCTR	0.164923	0.038263	0.291583	2.552063	0.010709
CRLF1	0.256893	0.058213	0.455574	2.534234	0.011269
RORC	−0.16456	−0.29418	−0.03494	−2.48825	0.012837
SEMA6C	0.232077	0.046502	0.417652	2.451102	0.014242
GH1	0.358254	0.067699	0.648809	2.416629	0.015665
C8G	0.164163	0.030955	0.297371	2.41542	0.015717
SLC11A1	0.159271	0.028752	0.28979	2.391725	0.016769
NRG1	−0.14474	−0.26347	−0.02601	−2.38925	0.016883
OXTR	0.179646	0.032036	0.327255	2.385344	0.017063
ULBP2	0.151162	0.026632	0.275691	2.379132	0.017353
IL17A	−0.14471	−0.26531	−0.02411	−2.35183	0.018681
STC2	0.138782	0.019326	0.258237	2.277062	0.022783
SLIT1	0.12663	0.017409	0.235851	2.27237	0.023064
JAG2	0.211641	0.026979	0.396304	2.246309	0.024684
BMP5	−0.09895	−0.1856	−0.0123	−2.23808	0.025216
NFATC1	0.170363	0.01987	0.320856	2.218741	0.026504
XCL1	0.136864	0.015786	0.257943	2.215501	0.026726
FABP4	0.08986	0.009572	0.170147	2.193642	0.028261
SCT	0.132533	0.013905	0.25116	2.18971	0.028545
EREG	−0.08382	−0.16025	−0.00739	−2.1495	0.031594
RBP7	0.164471	0.014072	0.31487	2.143349	0.032085
IL7	−0.16764	−0.32111	−0.01416	−2.14086	0.032286
AVPR2	0.145262	0.012182	0.278342	2.139384	0.032405
ULBP1	0.147132	0.01184	0.282423	2.131493	0.033049
UCN3	−0.10773	−0.20807	−0.00738	−2.10412	0.035368
ROBO3	0.181296	0.01165	0.350941	2.094562	0.03621
FLT4	0.179295	0.008545	0.350045	2.058043	0.039586
ORM1	−0.11875	−0.23203	−0.00547	−2.05453	0.039925
CXCL14	−0.08299	−0.16296	−0.00302	−2.0341	0.041942
CD1C	−0.14723	−0.28915	−0.00532	−2.0335	0.042002
CXCL1	−0.14131	−0.27823	−0.00439	−2.02283	0.043091
SEMA3E	0.093061	0.001024	0.185097	1.981781	0.047504
ADCYAP1R1	0.112046	8.25E‐04	0.223267	1.974505	0.048324
HSPA1L	0.245126	0.001523	0.488728	1.972217	0.048585
NGFR	0.107178	2.60E‐04	0.214096	1.964725	0.049446
NRG3	0.174329	1.13E‐04	0.348545	1.961238	0.049851

Abbreviations: HR, hazard rate ratio; OS, overall survival.

### 3.2 A prognostic model construction based upon immune genes

**FIGURE 2:**
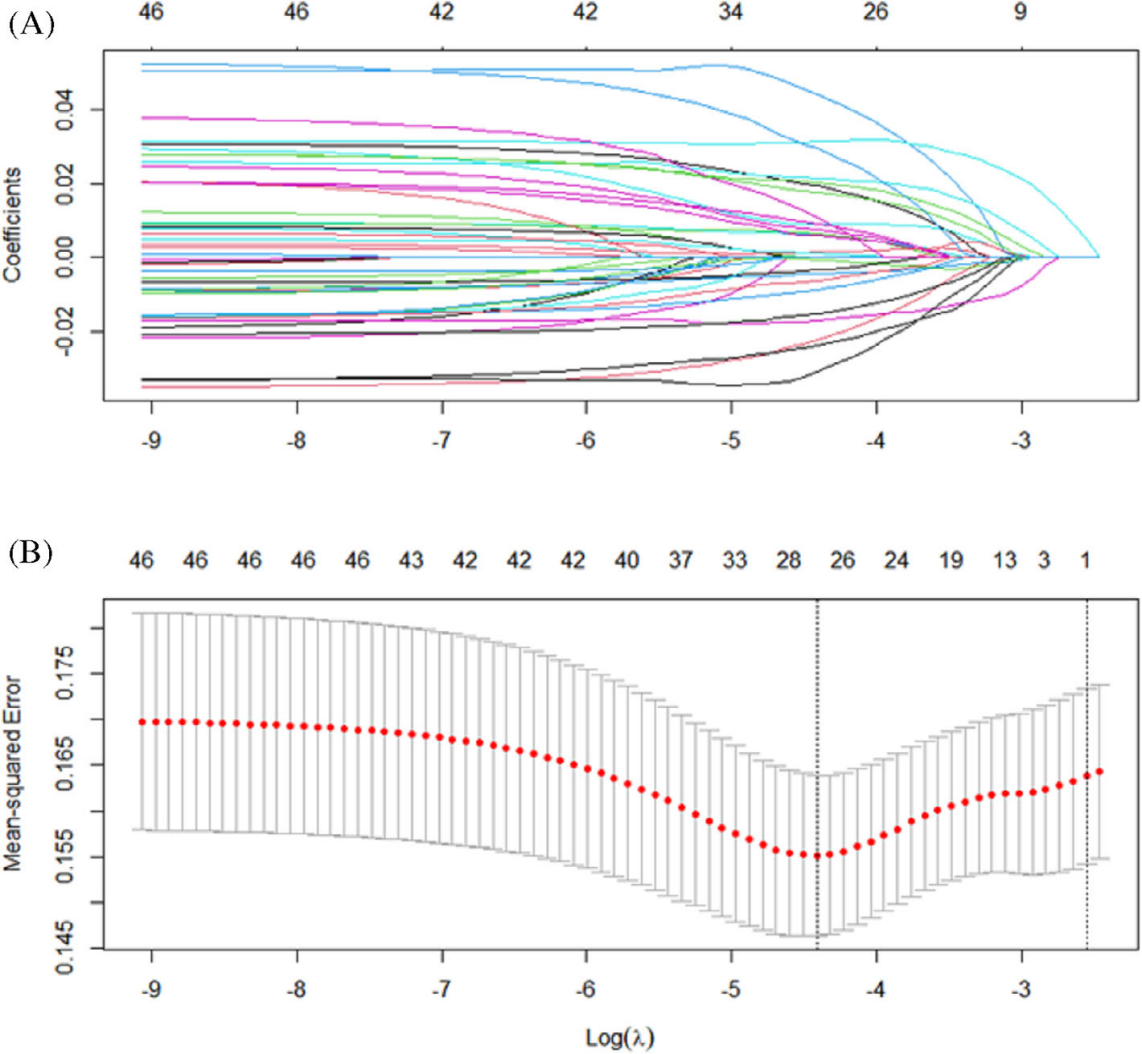
Establishment of a prognostic risk model by least absolute shrinkage and selection operator (LASSO) regression analysis. (A) LASSO coefficient profiles of the 46 genes in colon adenocarcinoma. (B) A coefficient profile plot was generated against the log (lambda) sequence. Selection of the optimal parameter (lambda) in the LASSO

LASSO Cox regression were performed to construct the prognostic risk model based upon the 46 candidate immune genes. Twenty‐six non‐zero coefficient genes were finally selected as key genes when the model reached the minimum value of *λ*. A risk model was then constructed with the 26 key genes (Figure 2A,B).

The immune score of each colon adenocarcinoma patient were calculated using the following formula:

$$\text{immune score}=\mathrm{SCT}*0.0651+\text{SCTR}*0.2110+\mathrm{IL}7*\left(-0.3138\right)+\text{ULBP}1*0.0825+\mathrm{IL}17A*\left(-0.0783\right)+\mathrm{STC}2*\left(0.1352\right)+\text{EREG}*\left(-0.0785\right)+\text{ULBP}2*0.0324+\mathrm{NGF}*\left(-0.0265\right)+\mathrm{XCL}1*\left(0.0813\right)+\text{RORC}*\left(-0.1411\right)+\text{CXCL}14*\left(0.0017\right)+\text{MCHR}2*0.1496+\mathrm{NRG}1*\left(-0.0562\right)+\mathrm{UCN}*0.1865+\text{FABP}4*\left(-0.0186\right)+\mathrm{LEP}*0.0364+C8G*0.0091+\mathrm{UCN}3*\left(-0.1071\right)+\text{OXTR}*\left(-0.0069\right)+\text{HSPA}1A*0.1246+\text{HSPA}1L*0.1010+\mathrm{ORM}1*\left(-0.1399\right)+\text{TDGF}1*\left(-0.0429\right)+\mathrm{NOX}5*0.1017+\mathrm{GH}1*0.3369$$

**FIGURE 3:**
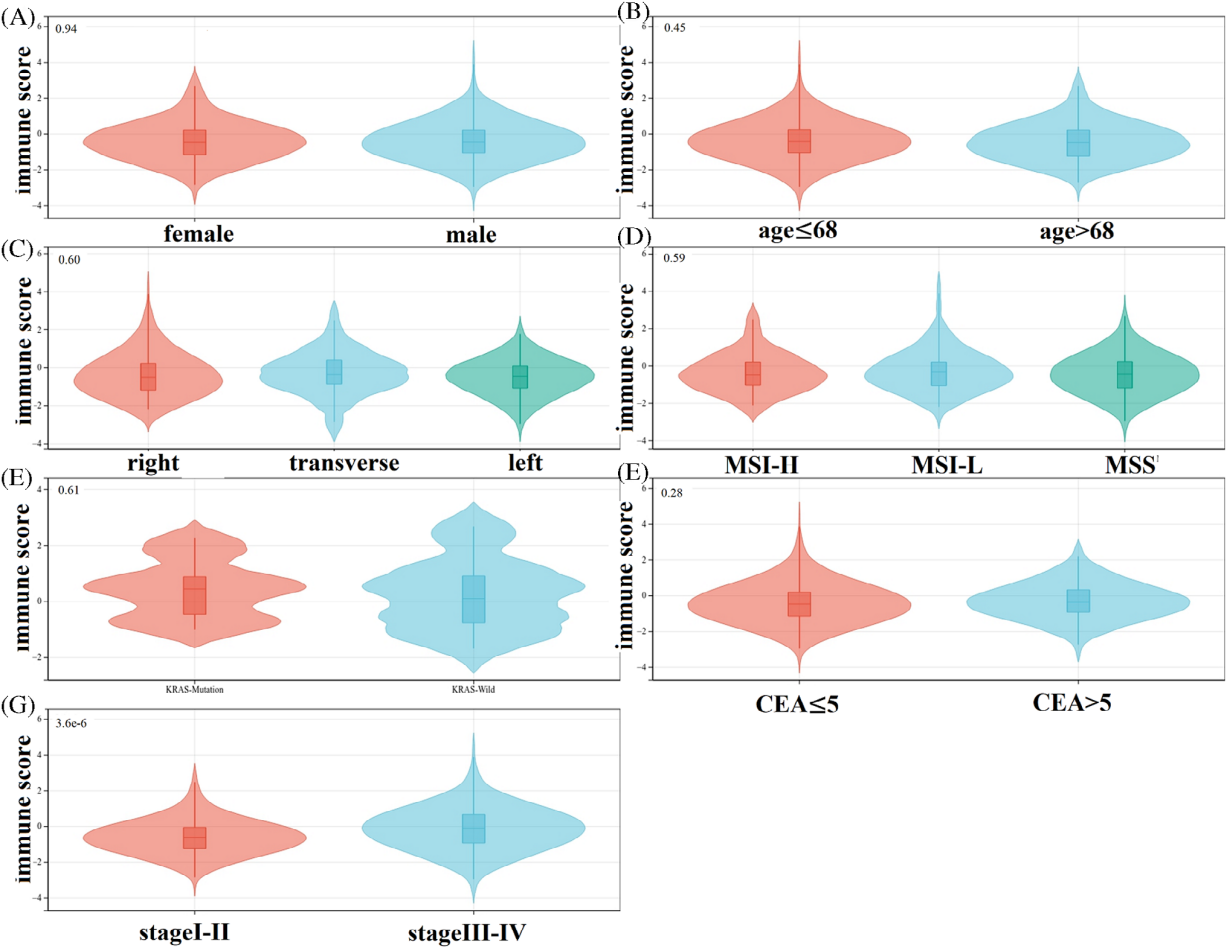
Correlation between immune scores and different clinical features. (A) Gender; (B) age; (C) primary tumor site; (D) microsatellite unstable state; (E) KRAS gene mutation state; (F) serum CEA level; (G) tumor‐lymph‐node‐metastasis stages

Figure 3 showed that the immune scores of the patients had no statistically relevant correlation with the following clinical features: gender (*p* = .94), age (*p* = .45), primary tumor site (*p* = .60), microsatellite unstable state (*p* = .59), the KRAS gene mutation state (*p* = .61), serum CEA level (*p* = .28). However, the immune scores tended to increase with the TNM stages (*p* < .01).

**FIGURE 4:**
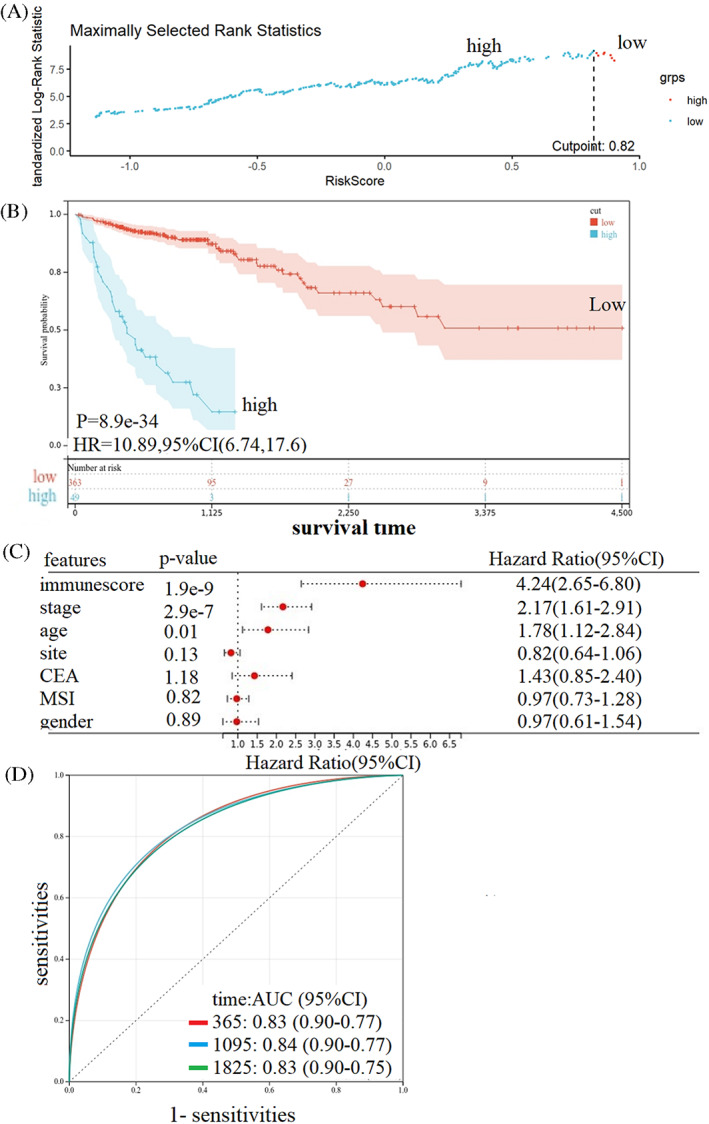
Immune scores and survival. (A) Cut‐off value of the immune scores for colon adenocarcinoma; (B) Kaplan–Meier curves of the OS for patients in the high‐ and low‐immune score groups; (C) multivariate COX regression analysis; (D) AUC of time‐dependent ROC curves verified the prognostic performance of the immune score model. AUC, area under the curve; OS, overall survival

The patients were then divided into a high immune score group (*n* = 363) and a low immune group (*n* = 49) based upon the optimal cut‐off value (immune score = 0.82, Figure 4A). Patients in high immune score group were shown to have significantly worse OS than those in low immune score group (HR = 10.89, 95% CI: 6.74–17.60, *p* < .01; Figure 4B). In addition, multivariate Cox regression result indicated that immune score was an independent prognostic for colon cancer (*p* < .01; Figure 4C). The time‐dependent ROC curves were performed to predict the performance of the immune score for colon adenocarcinoma patients' survival. The area under the curve (AUC) was 0.83 at 1 year, 0.84 at 3 years, and 0.83 at 5 years (Figure 4D).

**FIGURE 5:**
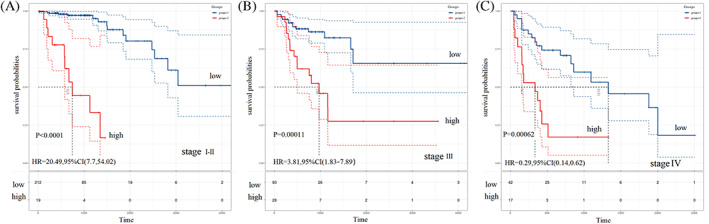
Kaplan–Meier curves of overall survival for patients with different stages, (A) stage I‐II, (B) stage III, (C) stage IV

The subgroup analyses showed that high immune score was a poor prognostic marker for colon adenocarcinoma patients with different TNM stage (Figure 5). For colon cancer patients diagnosed with early stages (stage I‐II), high immune score was associated with shorter survival compared with low immune score (HR = 20.49, 95% CI: 7.7–54.02, *p* < .0001, Figure 5A). For patients with stage III stage, high immune score was related with poor survival (high vs. low: HR = 3.81, 95% CI: 1.83–7.89, *p* = .00011; Figure 5B). Moreover, high immune score indicated a poor survival for patients with stage IV colon cancer (HR = 0.29, 95% CI: 0.14–0.62, *p* = .00062; Figure 5C).

### 3.3 Functional analyses for the immune genes

**FIGURE 6:**
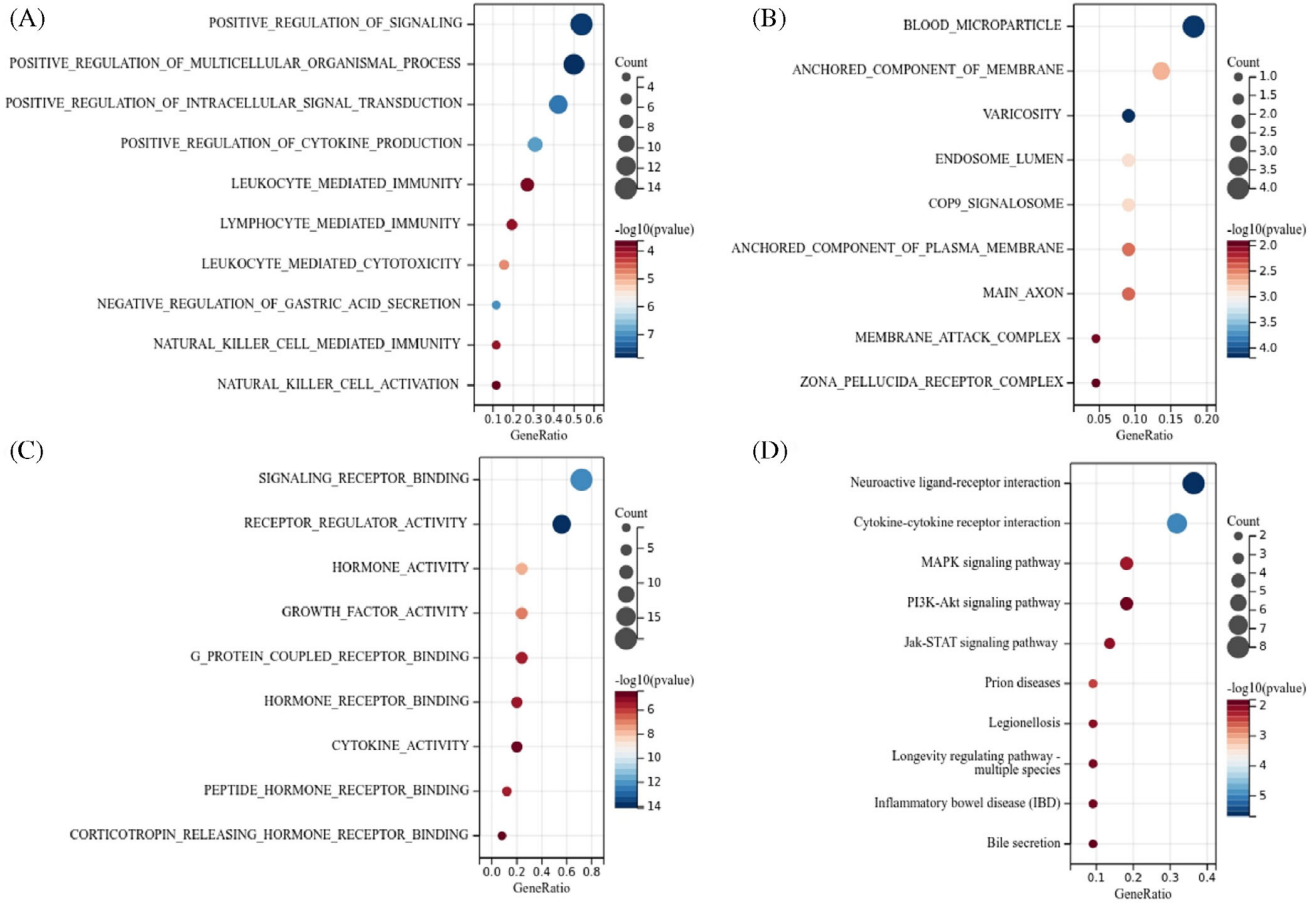
Functional enrichment analyses for the immune‐related genes in colon adenocarcinoma, (A) biological process, (B) cellular component, (C) molecular function, (D) Kyoto Encyclopedia of Genes and Genomes (KEGG) analysis

GO enrichment and KEGG pathway analyses were applied to further identify the biological functions and pathways of the immune genes with prognostic values. The immune genes were correlated to various immune‐related molecular functions, such as signal positive registration, cytokine‐mediated signal pathway, natural kill cell activation, leukocyte‐mediated cytotoxicity, lymphocyte‐mediated immunity, natural kill cell‐mediated immunity, natural killing cell activation, and so on (*p* < .05; Figure 6A). KEGG pathway analysis showed that these immune gene‐enriched pathways included natural killer cell‐mediated cytotoxicity, neuroactive ligand receptor interaction, helper T cell Th17 cell differentiation, and also cytokine receptor interaction pathways (*p* < .05; Figure 6D).

### 3.4 The Relationship between immune score and immune signals

**FIGURE 7:**
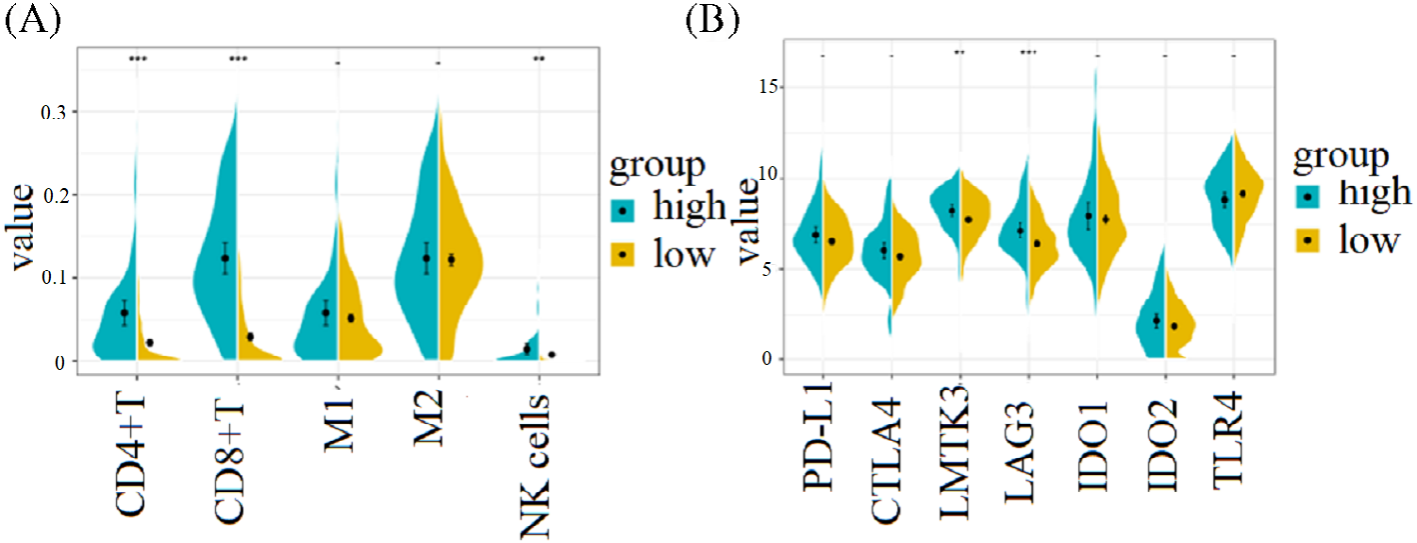
The relationship between immune score and immune signals, (A) immune score and immune cells, (B) immune score and immune escaping genes

According to CIBERSORT, the tumor tissues of patients in the high immune score group are rich in CD4+ T cells and CD8+ T cells (*p* < .001, Figure 7A). Meanwhile, there was no significant difference of macrophages and NK cells between the high and low immune score groups (Figure 7A).

Interestingly, we found that in the high immune score group, the expressions of LMTK3 and LAG3 were higher than those in the low score group (Figure 7B). The expression of PD‐L1 was also slightly higher in the high immune score group (*p* = .056; Figure 7B). And these genes played an important role in the immune escape process. It may be the reason why cancer cells are not subject to T cell attacks in an immunochemical environment.

### 3.5 The predictive value of immune score for treatments

Based on the results of the cMap database mapping, a total of the top 20 small molecule drugs were screened out, such as FGFR inhibitor, VEGFR inhibitors, TGF beta receptor inhibitor, EGFR inhibitor and so on (Table S1).

**FIGURE 8:**
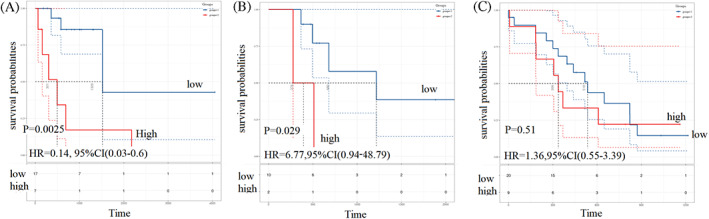
Kaplan–Meier analyses of progression‐free survival for patients receiving different chemotherapy. (A) fluorouracil monotherapy, (B) bevacizumab + fluorouracil + oxaliplatin combined therapy, (C) irinotecan + fluorouracil combined therapy

Kaplan–Meier analysis showed that for patients receiving fluorouracil monotherapy or bevacizumab + fluorouracil + oxaliplatin combination therapy, a high immune score was related to a shorter PFS (Figure 8A,B). However, no correlation was found between the immune score and PFS in patients receiving irinotecan chemotherapy (Figure 8C).

## 4 DISCUSSION

Colon adenocarcinoma is one of the world's most common cancers. The development and progression of colon adenocarcinoma is mainly caused by various genetic and epigenetic changes caused by abnormal gene expression.^11^ The immune system plays a vital role in the occurrence and development of tumors. In this study, a risk model of 26 prognostic immune genes was developed for colon adenocarcinoma patients using bioinformatics methods. High immune score was a poor prognostic marker for OS. Functional analyses showed that these immune genes were enriched during the immunization process.

Increasing evidence indicates that there is a correlation between cancer prognosis and the immune signature. Gan et al developed a three‐immune gene model for papillary thyroid carcinoma that includes HSPA1A, NOX5, and FGF23.^12^ Chen et al constructed an independent immune‐related genes prognostic model for breast cancer.^13^ And they found that high immune risk scores represented worse survival.^13^ Qiu et al identified an immune‐related gene‐based signature to predict prognosis of gastric cancer.^14^ They indicated that this immune signature could accurately distinguish patients into high‐ and low‐ risk groups.^14^ Compared with the previous studies, our analyses were sufficient and our results were also efficient. We found that a high immune score was related to a shorter PFS for patients receiving fluorouracil monotherapy or bevacizumab + fluorouracil + oxaliplatin combination therapy. This indicated practical value of the immune score in clinical work.

It was indicated that high immune score was related with poor survival for patients with colon adenocarcinoma. According to CIBERSORT, tumor sample with high immune score were enriched with CD4+ and CD8+ T cells. It is known that immunity plays a double‐edged sword in cancer. Immune system could distinguish cancer cells from normal ones via adaptive defense of immunity. At the same time, cancer cells can successfully escape the recognition of immune cells in several ways. The tumor microenvironment (TME) consists of cancer cells, stromal cells, chemokines and cytokines that can communicate through direct contact or secretion of relevant cytokines.^15^, ^16^ We found that the cancer cells could survive and develop a tumor, although the tumor samples with high immune scores were infiltrated with CD4+ T cells and CD8+ T cells. Chen et al developed and validated a five‐immune gene model of colon cancer. They found that the content of CD8+ T cells in colon cancer was decreased as the risk score increased. Their method of identifying key immune genes differed from ours. Their key genes were obtained from the training set (only 209 samples).^17^ However, we used the overall sample (*n* = 415) to obtain key genes. The immune score we constructed can also predict patient survival.

T cells are usually in the immunization monitoring state, which can only play only when they are activated. Complete activation of T cells rely on “double signal” system regulation: the first signal comes from its TCR (T cell receptor) and the specific binding of the complex of the antigen peptide–MHC (main tissue compatibility complex), that is, T cells are resistant to the original identification; the second signal comes from a synergistic stimulation molecule, that is, a synergistic stimulating molecules expressed by the antigen, the corresponding receptor or ligand interaction between the T cell surface, such as CD28/B7. In addition, in order to avoid the excessive stimulation of T cells, there is also a negative costimulatory molecule of T cells, mainly CTLA4‐B7 pathways and PD‐1/PD‐L1 pathways. Under normal circumstances, in order to prevent activated T cells from attacking normal human cells, the immune system can control the activation process of T cells by immune check points, and adjust the strength of the autoimmune response to maintain immune tolerance. Cancer cells mimic such procedures, suppress the activation of T cells and lead to immune escape.

Our result showed that several immune escape related signals on the cancer cell surface are significantly up‐regulated, including PD‐L1, LMTK3 and LAG3. These receptors are combined with ligands of the lymphocyte surface, making lymphocytes no longer identify cancer cells. This may explain the occurrence and development of tumors in an environment filled with a large amount of immune cells.

In addition, this study selected 20 small molecule drugs through the cMap. The commonly used drugs in clinical practice including FGFR inhibitor, VEGFR inhibitors, and EGFR inhibitor et al Increased evidences showed that FGFRs were the driving genes of various cancers, which were involved in the maintenance of the malignant characteristics of tumor cells in a “cell autonomy” manner and in the multiple steps of tumorigenesis and development.^18^ In recent years, many new drugs targeting FGFR have been in the clinical stage, such as AZD4547, FGF‐401, and ARQ087.^19^, ^20^ Anti‐VEGF‐VEGFR drugs roughly include the following categories: antibody drugs that directly target VEGF and VEGFR proteins, such as bevacizumab and ramucirumab; intracellular tyrosine kinase signaling pathway inhibitors, such as sorafi. There are also drugs such as fusion proteins and immunomodulators.^21^, ^22^ EGFR monoclonal antibodies (EGFRMAb), such as panitumumab and cetuximab, could inhibit tumor cell growth. And they have shown advantages in the treatment of stage IV colon cancer, either alone or in combination therapy with chemotherapy.^23^

Additionally, we assessed patient response to chemotherapy, and found that high immune score was related to poor clinical outcome of fluorouracil monotherapy or bevacizumab + fluorouracil + oxaliplatin combination therapy. However, due to the fact that the TCGA database did not provide immunotherapy information, we could not analyze the prognostic value of immune scores on immunotherapy‐treated patients. Therefore, further investigation should be conducted to explore the correlation between immune score and immunotherapy.

## 5 CONCLUSION

We developed a 26‐immune gene model of colon adenocarcinoma. This model could be used in clinical practice as a prognostic instrument for patients with colon adenocarcinoma. We developed a 26‐immune gene model of colon adenocarcinoma. This model could be used in clinical practice as a prognostic instrument for patients with colon adenocarcinoma.

## AUTHOR CONTRIBUTIONS

**Laiming Wei:** Conceptualization (equal); data curation (equal); formal analysis (equal); funding acquisition (equal); investigation (equal); methodology (equal); project administration (equal); resources (equal); software (equal); validation (equal); visualization (equal); writing – original draft (equal); writing – review and editing (equal). **Jing Xu:** Conceptualization (equal); data curation (equal); formal analysis (equal); investigation (equal); methodology (equal); resources (equal); software (equal); supervision (equal); validation (equal); visualization (equal); writing – original draft (equal); writing – review and editing (equal). **Xueyou Hu:** Funding acquisition (equal); writing – original draft (supporting); writing – review and editing (supporting). **Gang Lyu:** Conceptualization (equal); supervision (equal); writing – review and editing (supporting).

## CONFLICT OF INTEREST

The authors declare no conflict of interest.

## ETHICS STATEMENT

NA. Only the publicly available databases have been used in this study.

## Supporting information


**Appendix S1** Supporting Information

## Data Availability

Data sharing is not applicable to this article as no new data were created or analyzed in this study.
