# Supplementary material for: Development of a risk model based on immune genes in patients with colon adenocarcinoma
Source: Cancer Rep (Hoboken). 2022 Sep 4;6(2):e1712. doi: 10.1002/cnr2.1712 (PMC9939989; doi:10.1002/cnr2.1712)
Supplement: Supplementary file 1 — Appendix S1 Supporting Information [file CNR2-6-e1712-s001.docx]

**Supplemental Material for**

Development of a risk model based on immune genes in patients with colon adenocarcinoma

Laiming Wei^1^, Jing Xu^2*^, Xueyou Hu^1^, Gang Lyu^1,3*^

^1^Department of Electronic and Information Engineering, School of Advanced Manufacturing Engineering, Hefei University, Hefei 230601, China

^2^Department of Oncology, The First Affiliated Hospital of Anhui Medical University, Hefei 230022, China

^3^School of Big data and Artificial Intelligence, Chizhou University, Chizhou 247000, Anhui, China

*Corresponding authors: xujing@ahmu.edu.cn, lvgang@hfuu.edu.cn

**Section 1: Schoenfeld residuals test**

We took the HSPA1A gene as an example. First, we calculated the residuals of Cox regression for HSPA1A. Then, we performed a rank transformation on the survival time data in the original data. We made a scatter plot of the obtained residuals and practice ranks and plotted the total fitted line. The total fitted line fluctuated on the zero-scale line in general, that is, the survival risk did not change with time. Thus, we indicated that the effect of HSPA1A on the survival risk is in line with the PH equal proportion risk assumptions.


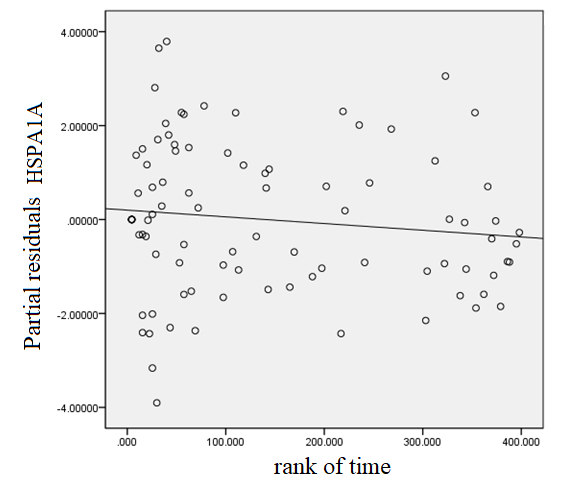


**Supplemental Figure 1.** Scatterplot of time rank and residuals of HSPA1A.

**Section 2: Regression analysis**

We performed regression analysis on immune score and survival time. The results of Durbin-Watson (D-W) test showed that D-W=2.161, which indicated immune score and survival time met the condition of linear regression independence.

Analysis of variance: F=6.521, P=0.011. It is indicated that immune score was a significant factor affecting patient survival.


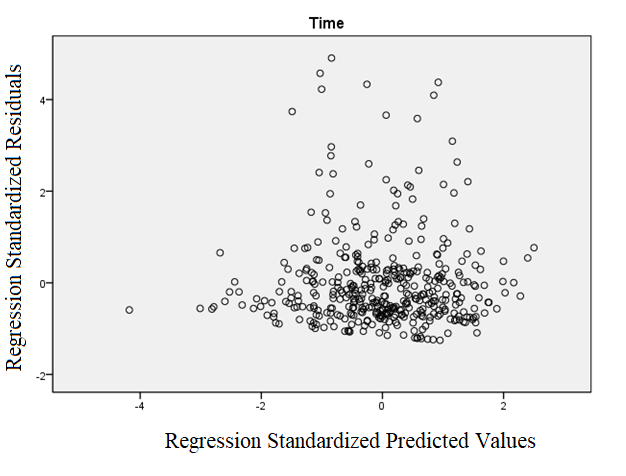


**Supplemental Figure 2.** Scatterplot of standardized residuals and standardized predicted values

The points in the scatterplot of standardized residuals and standardized predicted values are evenly distributed (**Supplemental Figure 2**). So, immune score and survival time might consider to meet the homogeneity of variance.


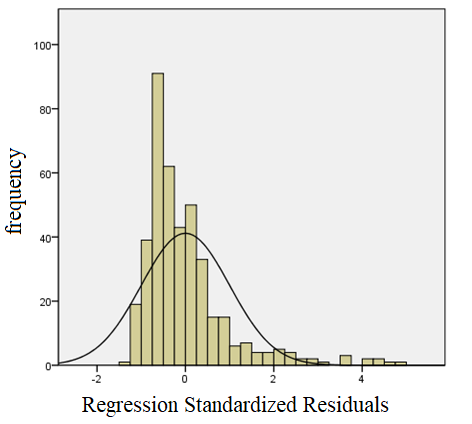


**Supplemental Figure 3.** Histogram of Standardized Residuals

As shown in **Supplemental Figure 3**, the standardized residuals are approximately normally distributed.


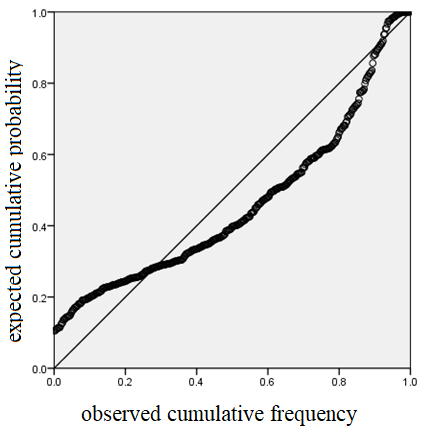


**Supplemental Figure 4.** P-P plot of residuals

Based on **Supplemental Figure 4**, the regression residuals were close to a normal distribution.

The overall tests showed that there was a linear relationship between immune-score and survival time.

**Section 3: Flow chart**

Colon cancer samples from TCGA database (n=459)

Excluded (n=44):

1.survival time less than 30 days or

2.insufficient clinical data

415 samples included

Divided in to 2 groups:

High-immune-score group

Low-immune-score group

Survival analyses (univariate Cox regression)

differential expression analyses

Gene enrichment

Immune DEGs

ROC curve

PFS of different treatments

cMAP analyses

key immune gene selection (LASSO Cox regression)

CIBERSORT algorithms

Immune score calculation

**Supplemental Figure 5**. Flow chart of our work. DEG: differential expression genes, PFS: progression-free survival.

**Section 4: cMap analysis**

**Supplemental Table 1**. the top 20 small molecule drugs potentially against colon adenocarcinoma.

| Name | Sensitive score |
| --- | --- |
| Sterol demethylase inhibitor | -99.26 |
| MDM inhibitor | -99.1 |
| Bacterial DNA gyrase inhibitor | -98.55 |
| FGFR inhibitor | -97.97 |
| Targets of VEGFR inhibitors GOF | -96.6 |
| TGF beta receptor inhibitor | -93.5 |
| Nucleoside reverse transcriptase inhibitor | -93.18 |
| Bacterial 30S ribosomal subunit inhibitor | -91.74 |
| HIV protease inhibitor | -89.33 |
| ATPase inhibitor | -88.26 |
| DNA synthesis inhibitor | -86.91 |
| Reverse transcriptase inhibitor | -86.78 |
| Protein synthesis inhibitor | -78.26 |
| HMGCR inhibitor | -74.35 |
| PARP inhibitor | -72.05 |
| Tubulin inhibitor | -70.71 |
| ATP synthase inhibitor | -47.79 |
| EGFR inhibitor | -47.76 |
| Cell Cycle Inhibition GOF | -47.62 |
